# A New Look at the Enterobacterial Common Antigen Forms Obtained during Rough Lipopolysaccharides Purification

**DOI:** 10.3390/ijms22020701

**Published:** 2021-01-12

**Authors:** Tomasz K Gozdziewicz, Anna Maciejewska, Alona Tsybulska, Czeslaw Lugowski, Jolanta Lukasiewicz

**Affiliations:** Laboratory of Microbial Immunochemistry and Vaccines, Ludwik Hirszfeld Institute of Immunology and Experimental Therapy, Polish Academy of Sciences, Weigla 12, 53-114 Wroclaw, Poland; tomasz.gozdziewicz@polpharmabiologics.com (T.K.G.); anna.maciejewska@hirszfeld.pl (A.M.); tsybulska26@gmail.com (A.T.); czeslaw.lugowski@hirszfeld.pl (C.L.)

**Keywords:** enterobacterial common antigen, ECA, cyclic ECA, ECA_PG_, lipopolysaccharide, LPS, mass spectrometry

## Abstract

Enterobacterial common antigen (ECA) is a conserved antigen expressed by enterobacteria. It is built by trisaccharide repeating units: →3)-α-D-Fuc*p*4NAc-(1→4)-β-D-Man*p*NAcA-(1→4)-α-D-Glc*p*NAc-(1→ and occurs in three forms: as surface-bound linear polysaccharides linked to a phosphoglyceride (ECA_PG_) or lipopolysaccharide − endotoxin (ECA_LPS_), and cyclic form (ECA_CYC_). ECA maintains, outer membrane integrity, immunogenicity, and viability of enterobacteria. A supernatant obtained after LPS ultracentrifugation was reported as a source for ECA isolation, but it has never been assessed for detailed composition besides ECA_CYC_. We used mild acid hydrolysis and gel filtration, or zwitterionic-hydrophilic interaction liquid (ZIC^®^HILIC) chromatography combined with mass spectrometry for purification, fractionation, and structural analysis of rough *Shigella sonnei* and *Escherichia coli* R1 and K12 crude LPS preparations. Presented work is the first report concerning complex characteristic of all ECA forms present in LPS-derived supernatants. We demonstrated high heterogeneity of the supernatant-derived ECA that contaminate LPS purified by ultracentrifugation. Not only previously reported *O*-acetylated tetrameric, pentameric, and hexameric ECA_CYC_ have been identified, but also devoid of lipid moiety linear ECA built from 7 to 11 repeating units. Described results were common for all selected strains. The origin of linear ECA is discussed against the current knowledge about ECA_PG_ and ECA_LPS_.

## 1. Introduction

Enterobacterial common antigen (ECA), described for the first time in the 1960s by Calvin M. Kunin [1], is a conserved antigen present in almost all Gram-negative bacteria belonging to Enterobacteriaceae family, such as *Escherichia coli*, *Shigella* spp., or *Klebsiella pneumoniae* [2,3]. Enterobacteria are causative agents of variety of infectious diseases, including intestinal and nosocomial infections with limited treatment in the case of multidrug resistant strains. ECA is heteropolysaccharide build by trisaccharide repeating unit: →3)-α-D-Fuc*p*4NAc-(1→4)-β-D-Man*p*NAcA-(1→4)-α-D-Glc*p*NAc-(1→[4] that is partially *O*-acetylated (OAc) at position 6 of the→4)-α-D-Glc*p*NAc-(1→ and a random distribution of free amino groups were reported for this residue in ECA_CYC_ [5,6]. ECA occurs in three different forms: as surface-bound linear polysaccharide linked to a phosphoglyceride (ECA_PG_), cyclic oligosaccharide composed of 3–6 trisaccharide subunits (ECA_CYC_) [5,7,8,9,10], and as polysaccharide linked to lipopolysaccharide (ECA_LPS_) [1,11,12]. ECA was discovered by observation of broad cross-reactivity between strains of *E. coli* causing urinary tract infections and rabbit antisera generated against the strains and 102 homologous and heterologous *E. coli* strains [1,3]. ECA_PG_ represents a major form of ECA and together with lipopolysaccharide (LPS) and ECA_LPS_, is located on the cell surface, contributing to antigenicity and outer membrane integrity. Serological observations suggested ECA_LPS_ as the only immunogenic form of ECA capable to generate anti-ECA antibodies upon immunization [1,3,11,12,13,14]. ECA_CYC_ is located in the periplasm and has been recently pointed out as an important factor maintaining the outer membrane permeability barrier [15]. Although the most conserved enterobacterial antigen ECA has been discovered over half century ago, in early 1960s, it still remains molecule of interest. Even though the presence of ECA_LPS_ form was suggested at stage of ECA discovery, the covalent linkage between ECA and LPS has been proven by relatively recent studies for *S. sonnei* phase II [11] and *E. coli* R1, R2 and R4 [12]. ECA coexistence with O-specific polysaccharide on the same LPS molecule within deep inner core region of *Yersinia enterocolitica* LPS was also suggested [16]. Finally the biological role of ECA_CYC_ was identified as osmotic sensor [17] and important factor maintaining the outer membrane permeability barrier [15]. Additionally, synthetic ECA oligosaccharides were obtained for the development of universal monoclonal antibody-based immunotherapy for drug-resistant enterobacteria [18].

Isolation and purification protocols of ECA differ depending on the ECA form and applied methodology of particular research group. The structure of ECA repeating unit came from studies of Männel and Mayer for *Salmonella enterica* serovar Montevideo who showed its two out of three sugar constituents [19] and Lugowski et al. for smooth *S. sonnei* phase I, where linear and *O*-acetylated complete trisaccharide ECA repeating unit was identified [4]. Männel and Mayer isolated ECA by combined phenol/water and phenol/chloroform/petroleum (PCP) ether extractions which led to final most likely ECA_PG_ isolation [19]. Lugowski et al. used ECA extracted with 85% ethanol from freeze dried supernatant obtained after bacterial mass sonication overnight in water at 100 °C followed by lysozyme treatment and ultracentrifugation [20]. The same ECA preparation was further examined using chemical methods and fast-atom-bombardment mass spectrometry (MS) showing finally its cyclic form composed from 4 to 6 repeating units, ECA_CYC_ [8]. Variety of ECA structures were reported based on described methodology of Männel and Mayer and Lugowski, where tetra-, penta-, and hexameric ECA_CYC_ prevailed as isolated and analyzed structures. In addition to initial studies of Männel and Mayer [19] and Lugowski et al. [4], the first indication for linear ECA resulted from serological observations of reactivity between monoclonal antibody against ECA and ECA preparations of various SDS-solubilized *E. coli* and *Salmonella enterica* serovar Typhimurium bacteria or ECA preparation thereof [21]. In cited study monoclonal antibody was generated by immunization of mice with formalized *E. coli* K12 and ECA was prepared according to the method of Männel and Mayer by combined phenol-water and PCP extraction of bacterial mass [19]. Immunoblotting of SDS-solubilized rough *E. coli* and *S. enterica* serovar Typhimurium bacteria with the anti-ECA monoclonal antibody demonstrated the ladder-like banding pattern of ECA molecules. The molecular weights of identified ECA forms was estimated with the use of molecular weight markers and ranged from about 10 to 35 or even 80 kDa. Similar observations were made by Kuhn et al. [22] and Böttger et al. [2] with the use of the same antibody for the screening of ECA preparations of various species belonging to the family Enterobacteriaceae. Kuhn et al. also suggested a lipid moiety (phosphoglyceride) of linear ECA and called this form ECA_PG_ and demonstrated that ECA preparations modified at its lipid moiety by phospholipases A2 and D or by mild acid hydrolysis lost its coating capacity, but still retain serological reactivity. Biological repeating unit was then identified as →3)-α-D-Fuc*p*4NAc-(1→4)-β-D-Man*p*NAcA-(1→4)-α-D-Glc*p*NAc-(1→by Kuhn et al. [23]. Further, trimeric (minor form), tetrameric (major form), and pentameric (minor form) ECA_CYC_ and low- and high-molecular weight linear ECA were identified by Vinogradov et al. in the ECA preparations of *Yersinia pestis*, where random distribution of free amino groups (GlcN) were identified [5]. This was the only report describing trimeric cyclic ECA. The authors used ECA isolated from bacterial cells with cold trichloroacetic acid followed by fractionation by gel-permeation and anion-exchange chromatography [5]. In addition to ECA_CYC_, linear ECA were observed by nuclear magnetic resonance (NMR) spectroscopy. Additionally, a crystal structure of tetrameric ECA_CYC_ was obtained for *Proteus penneri* strain 17 (1410–75), where ECA_CYC_ was isolated from LPS preparation degraded by 1% aqueous acetic acid and purified on a Sephadex G-50 column [24]. Additionally, a few reports utilized isolation of ECA directly from LPS supernatant obtained after LPS extraction without ethanol precipitation step. For example, Fregolino et al. have shown that supernatant obtained during ultracentrifugation of crude *E. coli* O157:H^−^ LPS contained mainly ECA_CYC_ composed of four and five repeating units fully N-acetylated and devoid of *O*-acetyl groups [25]. They have used enzymatic hydrolysis to digest the impurities (proteins and nucleic acid) which are present in the supernatant, then purified and fractionated supernatant by Sephacryl HR-100 and Bio-Gel P-100. Finally, we have demonstrated for *S. sonnei* phase II and *E. coli* R1, R2 and R4 the presence of ECA_LPS_ proving the covalent linkage between ECA and LPS and ECA biological repeating unit [11,12]. ECA_LPS_ is co-purified with other forms by methodology common for LPS—phenol-water extraction [26] and its polysaccharide part is isolated from LPS by mild acid hydrolysis and gel chromatography [11].

Studies of Gozdziewicz et al., besides ECA_LPS_ identification, showed coexistence of trace amounts of ECA_CYC_ and linear ECA in fractions collected during separation of poly- and oligosaccharides obtained from LPS degraded by mild-acid hydrolysis and purified by ultracentrifugation. Delipidated fractions of LPS and ECA_LPS_ of *E. coli* R1, R2, and R4 also contained highly heterogenic ECA_CYC_ and linear ECA coexisted with LPS-derived core oligosaccharides [12].

Observations gained by ECA_LPS_ analysis, particularly the presence of rarely reported linear ECA, in LPS-derived fractions of *S. sonnei* and *E. coli*, have prompted us into detailed analysis of the supernatant fraction obtained upon ultracentrifugation of LPS [12,25]. Moreover, even though linear ECA was reported in a few ECA preparation [5], it had never been characterized in details. The work of Fregolino et al. demonstrated rather simple composition since the authors focused on ECA_CYC_ purification. Thus, linear ECA seems to be the less characterized form of ECA in the supernatant and further studies on ECA composition are reasonable taking into account complete characteristics of this conserved antigen of enterobacteria. In order to provide the most native form of ECA and minimize selection pressure put on ECA isolation by ethanol precipitation or ion-exchange chromatography, we utilized herein modified protocol of Fregolino et al. [25] of supernatant purification by mild acid hydrolysis and centrifugation to remove denaturated proteins and nucleic acids, and finally Bio-Gel P-30 chromatography for ECA fractionation. In most reports ethanol precipitation resulted finally in ECA_CYC_ identification. As a result, we characterized in detail, composition of LPS-derived supernatant regarding ECA forms in rough *S. sonnei* phase II, *E. coli* R1 and K12. The reason behind the strain’s selection was broad experience gained from general ECA analysis in these strains. Moreover, all used strains are among first examples where ECA has been observed and studied for years, including ECA_CYC_ and ECA_LPS_ discovery [8,11,12]. Additionally, its roughness (the lack of the O-specific polysaccharides) facilitates structures elucidation. Electrospray ionization (ESI) MS was used for structural analysis of ECA and ESI-MS-coupled with a zwitterionic-hydrophilic interaction liquid chromatography (ZIC^®^HILIC) was tested for efficiency of separation of ECA forms according to the length, form, and acetylation level. An occurrence of cyclic and linear ECA characterized by various length and O/N-acetylation is reported for all LPS-derived supernatants.

## 2. Results

### 2.1. Supernatants Isolated upon LPS Ultracentrifugation Are Reach in Linear and Cyclic ECA

**Figure 1:**
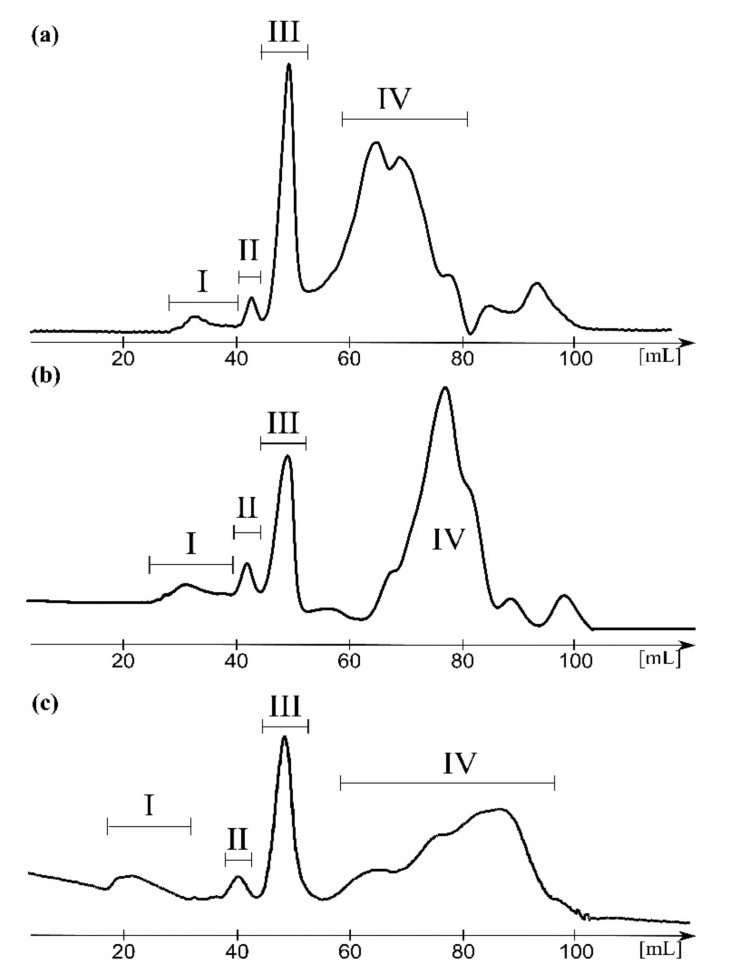
The Bio-Gel P-30 elution profiles of hydrolysed supernatants obtained after ultracentrifugation of *S. sonnei* phase II (**a**), *E. coli* R1 (**b**), and *E. coli* K12 (**c**) lipopolysaccharides (LPS). Fractions I, II, III, and IV were collected and further analyzed by mass spectrometry.

All *S. sonnei* phase II, *E. coli* R1, and K12 LPS-derived supernatants were hydrolyzed with 1.5% acetic acid in 100 °C for 1 h to degrade or denaturate nucleic acids and proteins and further purified by gel filtration chromatography on the Bio-Gel P-30 yielding from one to two high molecular weight fractions (I-II) and two low molecular weight fractions (III and IV) (Figure 1). Obtained fractions were analyzed by ESI-MS. Fractions I-III were attributed to various forms of ECA and analyzed below in details, whereas fractions IV contained residual tetrameric cyclic ECA and core oligosaccharide and Kdo-Hep fragments derived from hydrolyzed LPS present in trace quantities in all supernatants (data not shown).

**Figure 2:**
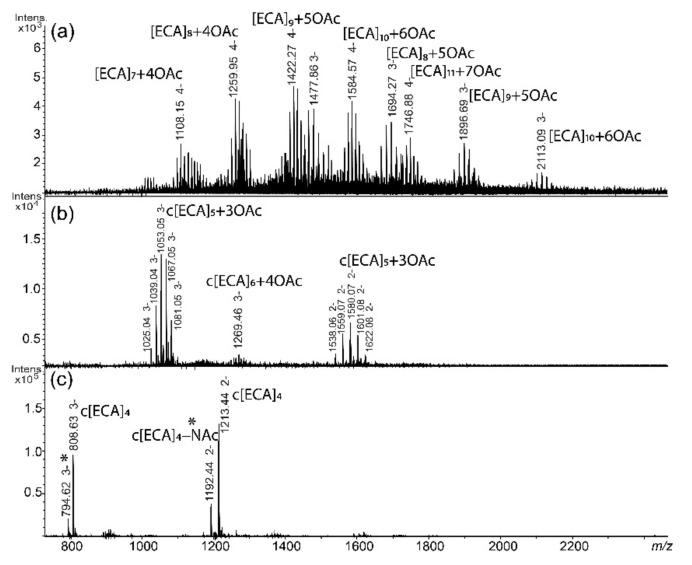
The ESI-MS spectra of the enterobacterial common antigen (ECA) forms obtained from *S. sonnei* phase II hydrolysed LPS-derived supernatant acquired in negative-ion mode for the (**a**) fraction I; (**b**) fraction II; and (**c**) fraction III. [ECA], trisaccharide repeating unit of ECA: →3)-α-D-Fuc*p*4NAc-(1→4)-β-D-Man*p*NAcA-(1→4)-α-D-Glc*p*NAc-(1→ present as constituent of linear [ECA]n or cyclic form of ECA c[ECA]n; OAc—*O*-acetyl group; c[ECA]_4_−NAc marked also by * stands for a c[ECA]_4_ where one of GlcNAc residues is replaced by GlcN [5,6]. Arabic numerals indicate charge of the ion.

For *S. sonnei* phase II, the ESI-MS spectrum of the fraction I isolated from supernatant revealed the presence of multiple negatively charged ions related to linear ECA polysaccharides—[ECA]_n_ with characteristic *m*/*z* values differences between ions corresponding to subsequent sugar residues of ECA as well as different number of *O*-acetyl groups—OAc (mass difference of 42.01 Da; *m*/*z* 10.50 difference for [M−4H]^4−^ ions, *m*/*z* 14.00 for [M−3H]^3−^ ions, and *m*/*z* 21.01 for [M−2H]^2−^ ions) (Figure 2a). The number of ECA trisaccharide repeating units in linear forms ranged from 7 to 11 according to the interpretation of *m*/*z* values based on the known ECA repeating unit structure (Table 1). The fraction II contained a cyclic form of ECA marked by c[ECA]_n_ symbol and constituted by five trisaccharide repeating units c[ECA]_5_ and small amount of c[ECA]_6_ (Figure 2b). Cyclic glycoforms characterized by different level of *O*-acetylation were also identified. The fraction III contained c[ECA]_4_ (Figure 2c). An interpretation of ions observed for *S. sonnei* supernatant is shown in Table 1, with assumption that all Glc*p*N residues in the ECA repeating unit are N-acetylated (Glc*p*NAc). A random distribution of free amino groups was reported for this residue that could not be distinguished by single-stage MS spectrum [5,6]. It means that the same *m*/*z* value corresponds to ions attributed to [ECA]_9_ + 5OAc and [ECA_GlcN_]_9_ + 4OAc, where one repeating unit of ECA polymer is devoid of one NAc group.

**Figure 3:**
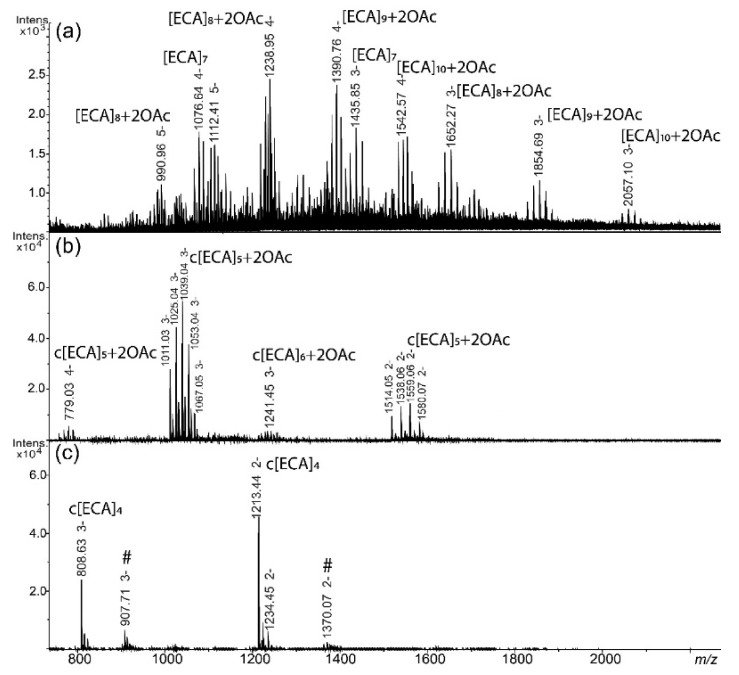
ESI-MS spectra of ECA forms obtained from *E. coli* R1 lipopolysaccharide (LPS) supernatant acquired in negative-ion mode for (**a**) fraction I; (**b**) fraction II; and (**c**) fraction III. [ECA], trisaccharide repeating unit of ECA: →3)-α-D-Fuc*p*4NAc-(1→4)-β-D-Man*p*NAcA-(1→4)-α-D-Glc*p*NAc-(1→ present as constituent of linear [ECA]_n_ or cyclic form of ECA c[ECA]_n_; OAc—*O*-acetyl group. Arabic numerals indicate charge of the ion; #—non-interpreted ions.

The hydrolysed supernatant of *E. coli* R1 showed similar elution profile as *S. sonnei* phase II and a composition that was determined by identical analytical protocol based on ESI-MS (Figure 3; Table 2).

The fraction I was characterized by the presence of linear polymers of ECA with characteristic *m*/*z* values differences between ions corresponding to OAc (Figure 3a). Linear *O*-acetylated ECA polysaccharides built from 7 to 10 repeating units were identified in fraction I of *E. coli* R1; however, lower number of OAc groups were identified for *E. coli* R1 in comparison with *S. sonnei* phase II. Fraction II contained in general ECA_CYC_ as c[ECA]_5_ and small amount of c[ECA]_6_ (Figure 3b), whereas the fraction III consisted of c[ECA]_4_. The cyclic forms of ECA in *E. coli* R1 were also substituted with several OAc groups (Figure 3b). An interpretation of ions is shown in Table 2.

**Figure 4:**
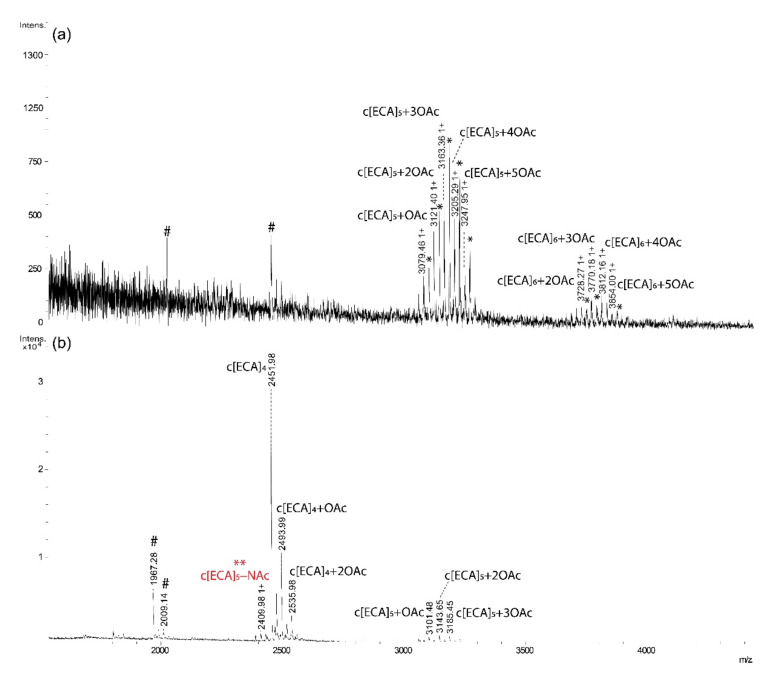
MALDI-TOF mass spectra of ECA forms obtained from *E. coli* K12 LPS supernatant acquired in positive ion mode for (**a**) fraction II; and (**b**) fraction III. [ECA], trisaccharide repeating unit of ECA: →3)-α-D-Fuc*p*4NAc-(1→4)-β-D-Man*p*NAcA-(1→4)-α-D-Glc*p*NAc-(1→present as constituent of linear [ECA]n or cyclic form of ECA c[ECA]n; Sodium adducts [M+H, Na]^+^ are marked by *; OAc—*O*-acetyl group; c[ECA]_4_−NAc marked also by ** stands for a c[ECA]_4_ where one of GlcNAc residues is replaced by GlcN [5,6]. Arabic numerals indicate charge of the ion; #—non-interpreted ions.

The hydrolyzed supernatant of *E. coli* K12 showed similar elution profile (Figure 1c) and composition as *S. sonnei* phase II and *E. coli* R1 (Figure 4, Table 3).

### 2.2. ECA Structure Verification by Mass Spectrometry

**Figure 5:**
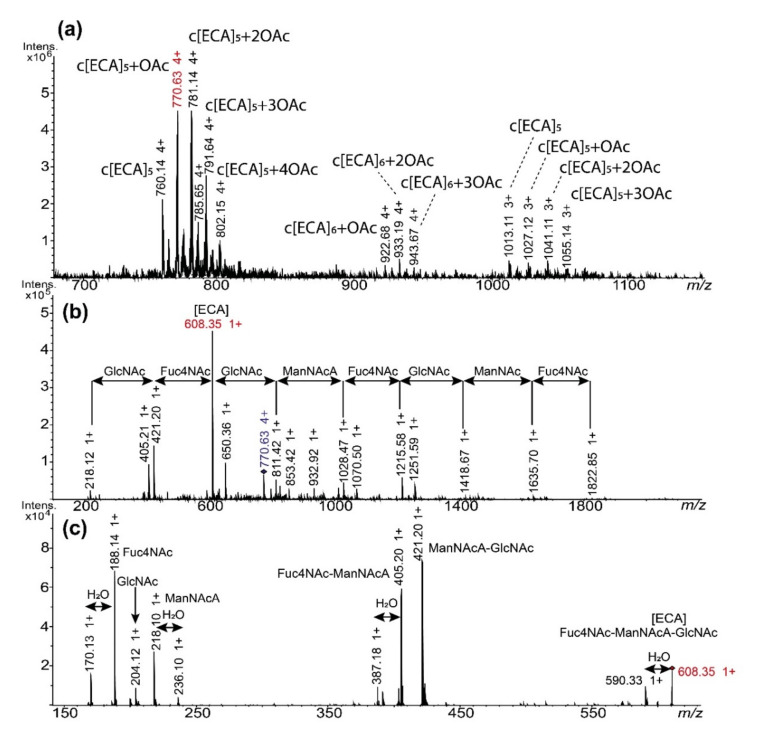
ESI-MS^n^ fragmentation analysis of c[ECA]_5_ isolated from *E. coli* R1 LPS supernatant. Spectra were acquired in positive-ion mode on ESI-IT mass spectrometer. (**a**) The MS spectrum of the fraction II; (**b**) The MS^2^ of the ion at *m*/*z* 770.63 (4+) ion corresponding to the c[ECA]_5_−OAc; (**c**) The MS^3^ of the ion at *m*/*z* 608.35 (1+) corresponding to one ECA repeating unit and resulted from the fragmentation of *m*/*z* 770.63→608.35. *m*/*z* values marked in red correspond to ions selected for MS^n^ fragmentation. GlcNAc, ManNAcA, and Fuc4NAc stand for ECA constituents; H_2_O stands for mass difference attributed to water molecule.

To confirm occurrence of ECA in supernatants we performed fragmentation analysis (ESI-MS^n^, positive-ion mode) for fraction II of *E. coli* R1 (c[ECA]_5-6_) (Figure 5). As expected, mass spectra of fraction (II) from *E. coli* R1 acquired in positive-ion mode were similar to those obtained in negative polarization, with mainly triple and quadruple protonated ions: [M + 3H]^3+^ and [M + 4H]^4+^ (Figure 5a). The ion with *m*/*z* 770.63 (4+) corresponding to c[ECA]_5_ with one OAc group was chosen for MS^n^ fragmentation (Figure 5b). The MS^2^ spectrum showed the ion series resulted mainly from glycosidic bonds fragmentation characterized by *m*/*z* differences corresponding to sugar residues from ECA repeating unit (Fuc4NAc—*m*/*z* 187.09, ManNAcA—*m*/*z* 217.06, GlcNAc—*m*/*z* 203.08). The most intense ion at *m*/*z* 608.35 was attributed to single ECA trisaccharide unit and was selected for further MS^3^ analysis (Figure 5c). Fragmentation of the ion at *m*/*z* 608.35 resulted in different disaccharide ions formation as well as particular, single charged, monosaccharide residues [M + H]^+^: *m*/*z* 188.14—Fuc4NAc, *m*/*z* 204.12—GlcNAc and *m*/*z* 218.10—ManNAcA. The fragmentation analysis confirmed the ECA presence in supernatant fractions.

### 2.3. ZIC^®^HILIC Fractionation of ECA Forms

**Figure 6:**
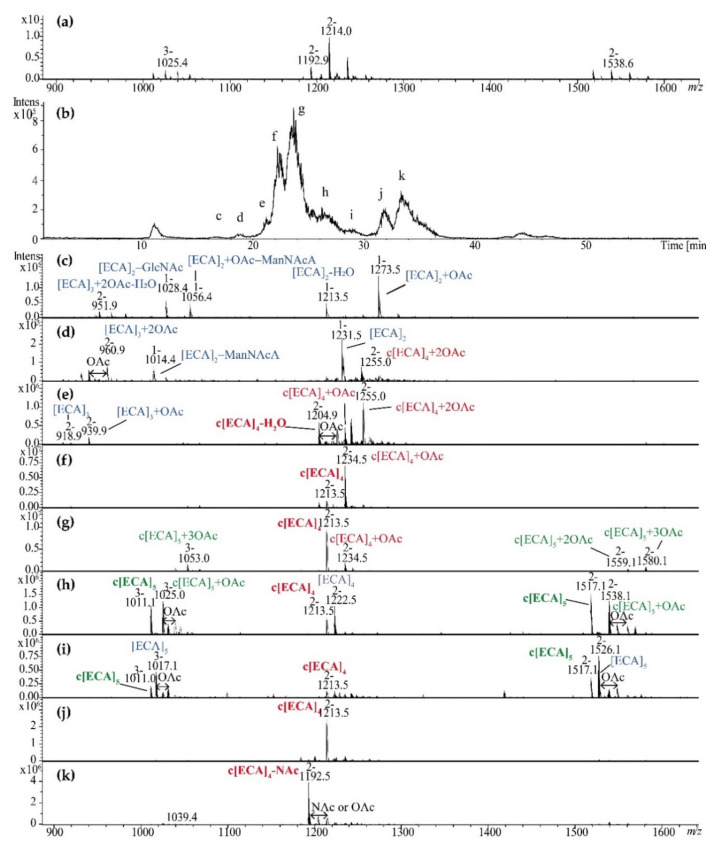
ZIC^®^HILIC LC-MS separation of *E. coli* R1 c[ECA]n (pooled fraction II and III isolated by Bio-Gel P-30 fractionation). (**a**) Negative ion mode ESI-MS spectrum of fractions II and III (pooled). (**b**) Base peak chromatogram (BPC) for fractionation in 70–40% gradient of acetonitrile in 0.1% formic acid. (**c**–**k**) MS scans corresponding to fractions as it was marked in the panel (**b**). [ECA], trisaccharide repeating unit of ECA: →3)-α-D-Fuc*p*4NAc-(1→4)-β-D-Man*p*NAcA-(1→4)-α-D-Glc*p*NAc-(1→present as constituent of linear [ECA]n or cyclic form of ECA c[ECA]n; OAc or NAc—O- or N-acetyl group; c[ECA]_4_−NAc stands for c[ECA]_4_ where one of GlcNAc residues is replaced by GlcN [5,6]. Arabic numerals indicate charge of the ion. Blue, red, and green colors mark linear ECA, c[ECA]_4_ + nOAc, and c[ECA]_5_ + nOAc respectively, where non *O*-acetylated c[ECA]_5_ and c[ECA]_4_ is marked by bold green and red font, respectively.

Since supernatant-derived ECA fractions exhibited high complexity due to the number of repeating units and OAc groups, ZIC^®^HILIC coupled with ESI-MS was examined for analytical fractionation of heterogenic fractions obtained from Bio-Gel P-30 (pooled fraction II and III) (Figure 6). Separation of pooled Bio-Gel P-30 II and III fractions resulted in nine c–k fractions (Figure 6b) further examined by ESI-MS (Figure 6c–k). Results demonstrated that the method may offer an efficient analytical and separation tool characterized by some limitations, especially to separate linear ECA (fractions c, d) from majority of c[ECA]_4-5_ (fractions f–k; marked in red). Examined method was not efficient to separate c[ECA]_5_ from the mixture of c[ECA]_4-5,_ however c[ECA]-NAc (fraction k) was separated in some extend from c[ECA]_4_ (fraction j) and its *O*-acetylated forms (fraction f). Obtained results demonstrated that ZIC^®^HILIC chromatography combined with ESI-MS may be utilized in separation of different forms of ECA. Additionally, contrary to Bio-Gel P-30 fractions, new structural details were identified, such as low-molecular weight linear ECA built of 2–5 repeating units (fractions c, d, e, h, I; marked in blue). Fraction c contained the ion at *m*/*z* 951.9 that might be attributed to dehydrated linear [ECA]_3_ + 2OAc or c[ECA] + 2OAc (Figure 6c).

## 3. Discussion

ECA structures, biosynthesis pathways, and biological role and immunogenicity are still in the field of interest for researchers. Studies on ECA require relatively pure and easy to obtain naturally occurred ECA antigen with defined structure. Despite of variety of reports on ECA isolation methodology and its final structural analyses described in the Introduction, there was still the need to explore complexity of various ECA preparations. LPS-derived supernatant as a source of ECA was such a case. In presented studies we have widen the knowledge about ECA heterogeneity and variability with its structural characteristics in the popular source for ECA isolation, a supernatant obtained after LPS ultracentrifugation. Our preliminary observations indicated higher complexity of the supernatant [11,12] than previous reports, including the work of Fregolino et al. [25]. We utilized herein simple protocol of supernatant purification by mild acid hydrolysis in 100 °C and centrifugation to remove denaturated proteins and nucleic acids followed by Bio-Gel P-30 gel chromatography for fractionation. The method has provided relatively native forms of ECA and minimized selection pressure put on ECA forms separation by ethanol precipitation (ECA_CYC_ prevailed in publications) or ion-exchange chromatography (ECA_CYC_).

For *S. sonnei* phase II, *E. coli* R1, and K12 linear ECA polysaccharides and cyclic ECA were identified, where linear ECA were detected as free polysaccharide without a lipid moiety. Linear ECA detected comprised from 7 to 10/11 (depending on strain) ECA repeating units characterized by high level of *O*-acetylation. Compared with *S. sonnei* phase II (4–9 *O*-acetyl groups), *E. coli* R1 showed lower *O*-acetylation level (0–5). Our MS analysis cannot exclude higher molecular weight polymers, since their MS detection might be hampered by low ionization potential. Described results were common for all selected strains, besides K12 strain and its fraction I where low quality spectra were obtained (data not shown). However, pattern of low resolution ions suggested the presence of linear ECA. It has to be emphasized that provided herein MS interpretation demanded *O*-acetylation of ECA form where all Glc*p* residues present in ECA repeating unit are N-acetylated (Glc*p*NAc). Since a random distribution of free amino groups was previously reported for this residue [5,6], the same *m*/*z* value might correspond to ions attributed to [ECA]_9_+5OAc or [ECA_GlcN_]_9_+4OAc, where one repeating unit of ECA polymer might be devoid of one NAc group. In regard to ECA_CYC_, only tetra-, penta-, and hexameric glycoforms were identified with different level of OAc groups. Obtained results are in agreement with the previous studies for *S. sonnei*, where for smooth strain also c[ECA]_4-6_ were observed [8]; however, our results provide detailed description of heterogeneity and *O*/*N*-acetylation levels that was not previously reported. *E. coli* R1 and K12 ECA_CYC_ forms resembled these of *S. sonnei* ECA. Generally tetrameric forms of ECA_CYC_ was nonacetylated in *S. sonnei* and *E. coli* R1. Contrary to Fregolino’s studies performed for smooth *E. coli* O157, non *O*-acetylated forms represents a minority of cyclic ECA in *S. sonnei* phase II and *E. coli* R1 and K12. According to our knowledge, this is the first report with detailed mass spectrometry data for all cyclic and linear forms of ECA in LPS-derived supernatant. No trimeric ECA_CYC_ were identified in selected strains, even in the fractions IV of the Bio-Gel P-30 chromatography. It is in agreement with most of previous studies besides single work of Vinogradov et al. for *Yersinia pestis* [5]. In the fraction c obtained by ZIC^®^HILIC chromatography we have found only one low abundant ion at *m*/*z* 951.9 that might be attributed both to dehydrated linear [ECA]_3_ + 2OAc or c[ECA]_3_ + 2OAc (Figure 6c). However the linear form is the most probable, since c[ECA]_3_ was reported only once [5], it was not detected among Bio-Gel P-30 fractions, and dehydrated ions are common for poly- and oligosaccharides. Final explanation requires pure sample and analysis performed by NMR spectroscopy to confirm the presence or lack of terminal residues.

We demonstrated that linear ECA devoid of lipid moiety constituted significant component of the supernatant, however the origin of linear ECA requires further discussion and future studies. Taking into account two known linear forms of ECA, ECA_PG_ and ECA_LPS_, these linear polysaccharides derived most probably from ECA_PG_. ECA_LPS_ has rather low impact on linear ECA generation, since it constitutes rare and low abundant form of ECA. For example, in *S. sonnei* phase II ECA_LPS_ substituted coexists as 3% of the total amount of poly- and oligosaccharides released after mild acid hydrolysis of LPS, indicating that unsubstituted core oligosaccharides prevail on the bacterial surface [11]. Moreover, our studies of ECA_LPS_ in *S. sonnei* and *E. coli* demonstrated stability of the covalent linkage between ECA and core oligosaccharide upon mild acid hydrolysis [11,12]. The linear ECA polysaccharides in obtained supernatants are derived most probably from ECA_PG_ form, which is co-extracted with LPS during hot phenol-water isolation method [27]. However, some doubts have appeared taking into account suggested chemical nature of the linkage between ECA and PG. The only reports on ECA and PG linkage are based on partial structural data. They postulated that the polysaccharide chains are covalently linked to diacyl glycerol through phosphodiester linkage [9,23]. The chemical nature of the linkage precludes acid lability in mild acid conditions under 100 °C. Thus, the only reason for linear ECA in mass spectra is an “in-source fragmentation” of ECA_PG_ polysaccharide part or other unknown kind of the linkage between ECA and PG.

Considering separation efficiency, the Bio-Gel P-30 fractionation was capable to separate linear ECA (fractions I) from *O*-acetylated mixture of c[ECA]_5-6_ (fractions II) and c[ECA]_4_ (fractions III). The fractionation still provides heterogeneous material characterized by nonstoichiometric and variable length and *O*/*N*-acetylation. Regarding the ZIC^®^HILIC, the HILIC was commonly used in glycomics for analysis of hydrophilic and polar compounds. The HILIC combined with zwitterionic stationary phase covalently attached to porous silica (ZIC^®^HILIC] was used as a tool to change the selectivity or to improve peak resolution for polar and hydrophilic compounds such as carbohydrates, metabolites, acids and bases, organic and inorganic ions, metal complexes, amino acids, peptides, and protein digests [28]. It seems to be a powerful technique in large-scale glycomics and glycoproteomics, such as the analysis of entire glycoproteomes at the glycopeptide level. We have also demonstrated its separation efficiency for LPS-derived core oligosaccharides [29]. Examination of the ZIC^®^HILIC results for ECA fractions, demonstrated that the method allowed to separate in some extend linear ECA (fractions c, d) from majority of c[ECA]_4-5_ (fractions f–k). Additionally, contrary to Bio-Gel P-30 fractions (II and III), low-molecular weight linear ECA built of 2–5 repeating units were observed during ZIC^®^HILIC separation. c[ECA]-NAc (fraction k) was separated in some extend from c[ECA]_4_ (fraction j) and its *O*-acetylated forms (fraction f) and all these fractions revealed purity enough for future NMR analysis or biological studies. Examined method was not efficient to separate c[ECA]_5_ from the mixture of c[ECA]_4-5_; however, further eluent optimization may enhance selectivity.

The presented work is the first report about complex characteristic of ECA forms present in *S. sonnei* and *E. coli* LPS-derived supernatant. Preparation and fractionation methodology has allowed for detection of all ECA glycoforms present in the supernatant to have a broad view of sample complexity. According to current knowledge of ECA forms, the selected source of ECA should reflect all naturally occurred forms. Even though the supernatant was well characterized particularly for the presence of ECA_CYC,_ presented studies provide complete description of polymerization and *O*-acetylation level. We have showed that the LPS-derived supernatant also contained heterogeneous linear ECA polysaccharides and both forms are characterized by high level of O- and N-acetylation. We have confirmed existence of tetra, penta-, and hexameric structures of ECA_CYC_ and provide data for further research on the origin of linear ECA forms and chemical nature of lipid moiety of ECA_PG_. However, the presence of naturally occurred linear ECA cannot be excluded.

## 4. Materials and Methods

### 4.1. Bacterial Strains

Rough strain *S. sonnei* phase II (PCM 1985) was obtained from the Polish Collection of Microorganism (PCM) at the Ludwik Hirszfeld Institute of Immunology and Experimental Therapy, Polish Academy of Sciences (Wroclaw, Poland). *E. coli* R1 (strain F470, a derivative of O8:K27-) was kindly donated by prof. Helmut Brade from Research Center Borstel in Borstel, Germany. *E. coli* K12 (rough mutant of strain W3110) was kindly donated by prof. Miguel A. Valvano from the Center for Infection and Immunity in Queen’s University Belfast, Belfast, Northern Ireland. Bacteria were grown in LB medium in 9 L fermenter (BioFlow 415, New Brunswick^TM^, Eppendorf, Inc., Framingham, MA, USA) as previously described [11]. After growing to logarithmic phase bacteria were phenol-killed (final phenol concentration 0.5%, *v*/*v*), harvested by flow centrifugation (36,000 rpm; CEPA, Carl Padberg Zentrifugenbau GmbH, Lahr, Germany) and lyophilized.

### 4.2. LPS Preparation

LPS was extracted from lyophilized bacteria by hot phenol–water method described by Westphal et al. [26]. The water phase was intensively dialyzed against deionized water for 3 days (ZelluTrans, 30 kDa MWCO; Carl Roth GmbH + Co., Karlsruhe, Germany) and lyophilized. The crude LPS was resuspended in ultrapure water, homogenized by sonication, and separated by threefold ultracentrifugation, each for 6 h at 105,000× *g* using Beckman Coulter centrifuge (Beckman Coulter Life Sciences Division, Indianapolis, IN, USA). The supernatant after first ultracentrifugation was collected and lyophilized, where LPS pallet was further purified according to need.

### 4.3. Isolation of ECA Forms

The supernatant from first ultracentrifugation (200 mg) were hydrolysed in 1.5% acetic acid (40 mL) in water bath, 100 °C for 1 h. After cooling the hydrolysate was centrifuged (40,000× *g*, 20 min) and resulted so called “secondary supernatant” was collected and freeze-dried. The pellet contained nucleic acids, proteins, and small amounts of lipids from LPS (lipid A) was discarded. Pre-purified supernatant (100 mg) was fractionated by gel filtration chromatography on Bio-Gel P-30 column (45–90 µm, 1.8 × 90 cm; Bio-Rad, Hercules, CA, USA) equilibrated with 50 mM pyridine-acetic acid buffer (pH 5.6) connected to differential refractometric detector (Knauer Wissenschaftliche Geräte GmbH, Berlin, Germany). The 1.5 mL fractions were collected, pooled, and lyophilized.

### 4.4. Mass Spectrometry

The ESI mass spectra were acquired on high resolution ESI-Q-TOF (electrospray ionization quadrupole time of flight) maxis impact (Bruker Daltonik GmbH, Bremen, Germany) in negative-ion mode with 200–2000 *m*/*z* scan range. External calibration of mass spectrometer was performed using ESI Tuning Mix (Agilent Technologies, Santa Clara, CA, USA) in negative-ion mode before analysis. Poly- and oligosaccharides were dissolved in acetonitrile/water (50:50 (*v*/*v*), 50 µg/mL) and directly injected to ESI source at 3 µL/min flow speed using syringe pump. The source parameters were as follow: source temp.: 200 °C; nitrogen flow, 5 l/min at a pressure of 8 psi.

Fragmentation analysis (MS/MS) was carried out on ESI-IT (ion trap) amaZon SL (Bruker Daltonik GmbH, Bremen, Germany) in positive ion mode. Oligosaccharide fraction was dissolved in acetonitrile/water/formic acid (50:50:0.5; 100 µg/mL). MS^n^ experiments were acquired in the 100–2000 *m*/*z* range using an isolation window of 4 *m*/*z*, an amplitude value of 0.35, and SmartFrag mode of 60–120%.

For ZIC^®^HILIC-ESI-MS chromatography poly- and oligosaccharide mixtures were loaded on SeQuant^®^ ZIC^®^HILIC semi-preparative (5 μm, 200 Å, 150 × 21.2 or 250 × 10 mm) column (HILICON AB, Umeå, Sweden). The columns were operated using Dionex UltiMate 3000 chromatography system (Thermo Fisher Scientific, Waltham, MA, USA) coupled to ESI mass spectrometer amaZon SL (Bruker Daltonik GmbH, Bremen, Germany). For separation of *E. coli* R1 ECA, 1 mg of sample (1 mg/mL in 70% ACN) was fractionated using two solvents: solvent A—acetonitrile, solvent B—0.1% formic acid with 70–40% gradient of A (50 min) at flow rate 2 mL/min. ESI source parameters were as follows: sample flow, 3 μL/min; ion source temperature, 200 °C; nitrogen flow, 5 μL/min at a pressure of 8 psi. Spectra were scanned in the 200–2000 *m*/*z* range. The system was calibrated using SI-L Tuning Mix (Agilent Technologies, Santa Clara, CA, USA).

MALDI-TOF mass spectra were acquired on UltrafleXtreme instrument (Bruker Daltonik GmbH, Bremen, Germany) in positive-ion mode. External calibration of mass spectrometer was performed using peptide or protein calibration standards (Bruker Daltonik GmbH, Bremen, Germany). Poly- and oligosaccharides were dissolved in water (0.5 mg/mL) and mixed with THAP or DHB matrix solution (10 mg/mL), dried in room temperature, and analyzed.

Obtained spectra were deconvoluted and analyzed in Data Analysis 4.0 or Flex Analysis software (Bruker Daltonik GmbH, Bremen, Germany). Fragmentation spectra were evaluated with assistance of GlycoWorkbench softwere [30].

## 5. Conclusions

Supernatant from ultracentrifugation of enterobacterial LPS is a rich source of several ECA forms like cyclic or linear polysaccharides. The *S. sonnei* phase II and *E. coli* R1 produce cyclic form of ECA build by 4 to 6 trisaccharide repeating units and linear ECA polysaccharides of altered chain length and *O*-acetylation profile. The *E. coli* K12 produced cyclic form of ECA build by 4 to 6 trisaccharide repeating units, similarly to *E. coli* R1 and *S. sonnei* phase II, however it demonstrated higher level of *O*-acetylation (from 1 to 7 *O*-acetyl groups per molecule). The origin of linear ECA requires further explanation, to confirm ECA_PG_ as a source of these glycoforms. Presented ECA isolation and purification method can be applied during further studies on ECA structural variability in other species of Enterobacteriaceae. ZIC^®^HILIC method seems to be useful tool for separation of some glycoforms of c[ECA]_n_.

## Figures and Tables

**Table 1:** Interpretation of main ions observed in fractions I–III from *S. sonnei* phase II supernatant (ESI-MS, negative ion mode, [M−nH]^n−^ ions).

Fraction	Polysaccharide Composition	Observed Ion (*m*/*z*)	Calculated Ion (*m*/*z*)	Theoretical Monoisotopic Mass (Da)	Interpretation of the Ion
I	[ECA]_7_ + 4OAc	1108.15	1108.15	4436.61	[M-4H]^4−^
		1477.86	1477.86		[M-3H]^3−^
	[ECA]_8_ + 4OAc	1259.95	1259.95	5043.83	[M-4H]^4−^
	[ECA]_8_ + 5OAc	1694.27	1694.27	5085.84	[M-3H]^3−^
	[ECA]_9_ + 5OAc	1422.27	1422.26	5693.07	[M-4H]^4−^
		1896.69	1896.68		[M-3H]^3−^
	[ECA]_10_ + 6OAc	1584.57	1584.57	6342.30	[M-4H]^4−^
		2113.09	2113.09		[M-3H]^3−^
	[ECA]_11_ + 7OAc	1746.88	1746.88	6991.53	[M-4H]^4−^
II	c[ECA]_5_ + 3OAc	1053.05	1053.04	3162.14	[M-3H]^3−^
		1580.07	1580.06		[M-2H]^2−^
	c[ECA]_6_ + 4OAc	1269.46	1269.45	3811.37	[M-3H]^3−^
III	c[ECA]_4_	808.63	808.62	2428.89	[M-3H]^3−^
		1213.44	1213.44		[M-2H]^2−^
	c[ECA]_4_ − NAc	794.62	794.62	2386.87	[M-3H]^3−^
		1192.44	1192.43		[M-2H]^2−^

[ECA], trisaccharide repeating unit of ECA: →3)-α-D-Fuc*p*4NAc-(1→4)-β-D-Man*p*NAcA-(1→4)-α-D-Glc*p*NAc-(1→present as constituent of linear [ECA]_n_ or cyclic form of ECA c[ECA]_n_; OAc—*O*-acetyl group; c[ECA]_4_−NAc stands for a c[ECA]_4_ where one of GlcNAc residues is replaced by GlcN [5,6].

**Table 2:** Interpretation of main ions observed in fractions I-III from *E. coli* R1 supernatant (ESI-MS, negative ion mode, [M-nH]^n−^ ions).

Fraction	Polysaccharide Composition	Observed Ion (*m*/*z*)	Calculated Ion (*m*/*z*)	Theoretical Monoisotopic Mass (Da)	Interpretationof the Ion
I	[ECA]_7_	1076.64	1076.64	4310.58	[M-4H]^4−^
		1435.85	1435.85		[M-3H]^3−^
	[ECA]_8_ + 2OAc	990.96	990.96	4959.81	[M-5H]^5−^
		1238.95	1238.95		[M-4H]^4−^
		1652.27	1652.26		[M-3H]^3−^
	[ECA]_9_ + 2OAc	1112.41	1112.40	5567.03	[M-5H]^5−^
		1390.76	1390.75		[M-4H]^4−^
		1854.69	1854.67		[M-3H]^3−^
	[ECA]_10_ + 2OAc	1542.57	1542.56	6174.26	[M-4H]^4−^
		2057.10	2057.08		[M-3H]^3−^
II	c[ECA]_5_ + 2OAc	779.03	779.02	3120.13	[M-4H]^4−^
		1039.04	1039.04		[M-3H]^3−^
		1559.06	1559.06		[M-2H]^2−^
	c[ECA]_6_ + 2OAc	1241.45	1241.44	3727.35	[M-3H]^3−^
III	c[ECA]_4_	808.63	808.62	2428.89	[M-3H]^3−^
		1213.44	1213.44		[M-2H]^2−^

[ECA], trisaccharide repeating unit of ECA: →3)-α-D-Fuc*p*4NAc-(1→4)-β-D-Man*p*NAcA-(1→4)-α-D-Glc*p*NAc-(1→present as constituent of linear [ECA]_n_ or cyclic form of ECA c[ECA]_n_; OAc—*O*-acetyl group; c[ECA]4−NAc stands for a c[ECA]_4_ where one of GlcNAc residues is replaced by GlcN [5,6].

**Table 3:** Interpretation of main ions observed in fractions II–III from *E. coli* K12 supernatant (MALDI-TOF MS, positive ion mode).

Fraction	Polysaccharide Composition	Observed Ion (*m*/*z*)	Calculated Ion (*m*/*z*)	Theoretical Monoisotopic Mass (Da)	Interpretation of the Ion
II	c[ECA]_5_ + OAc	3079.46	3079.13	3078.12	[M + H]^+^
	c[ECA]_5_ + 2OAc	3121.40	3121.14	3120.13	[M + H]^+^
	c[ECA]_5_ + 3OAc	3163.36	3163.15	3162.14	[M + H]^+^
	c[ECA]_5_ + 4OAc	3205.29	3205.16	3204.15	[M + H]^+^
	c[ECA]_5_ + 5OAc	3247.95	3247.17	3246.16	[M + H]^+^
	c[ECA]_6_ + 2OAc	3728.27	3728.35	3727.35	[M + H]^+^
	c[ECA]_6_ + 3OAc	3770.18	3770.37	3769.36	[M + H]^+^
	c[ECA]_6_ + 4OAc	3812.16	3812.38	3811.37	[M + H]^+^
	c[ECA]_6_ + 5OAc	3854.00	3854.39	3853.38	[M + H]^+^
III	c[ECA]_4_ − NAc *	2409.98	2409.86	2386.87	[M + H, Na]^+^
	c[ECA]_4_	2451.98	2451.87	2428.89	[M + H, Na]^+^
	c[ECA]_4_ + OAc	2493.99	2493.88	2470.90	[M + H, Na]^+^
	c[ECA]_4_ + 2OAc	2535.98	2535.90	2512.91	[M + H, Na]^+^
	c[ECA]_5_ + OAc	3101.48	3101.11	3078.12	[M + H, Na]^+^
	c[ECA]_5_ + 2OAc	3143.65	3143.12	3120.13	[M + H, Na]^+^
	c[ECA]_5_ + 3OAc	3185.45	3185.13	3162.14	[M + H, Na]^+^

[ECA], trisaccharide repeating unit of ECA: →3)-α-D-Fuc*p*4NAc-(1→4)-β-D-Man*p*NAcA-(1→4)-α-D-Glc*p*NAc-(1→present as constituent of linear [ECA]_n_ or cyclic form of ECA c[ECA]_n_; OAc—*O*-acetyl group; *—c[ECA]_4_−NAc stands for a c[ECA]_4_ where one of GlcNAc residues is replaced by GlcN [5,6].

## Data Availability

Data (unprocessed mass spectra) available on request from the corresponding author. The data are not publicly available due to presentation of all relevant chromatograms and mass spectra in the publication.

## Abbreviations

ECA	Enterobacterial common antigen
c[ECA]_n_	Cyclic ECA composed of n repeating units
[ECA]_n_	Linear ECA composed of n repeating units
ECA_PG_	Phosphoglyceride-linked ECA
ECA_CYC_	Cyclic ECA
ECA_LPS_	Lipopolysaccharide-associated ECA
ESI-IT MS	Electrospray ionization-ion trap mass spectrometry
LPS	Lipopolysaccharide
MALDI-TOF MS	Matrix-assisted laser-desorption/ionization-time of flight mass spectrometry
MS	Mass spectrometry
NAc	*N*-acetyl group
NMR	nuclear magnetic resonance (spectroscopy)
OAc	*O*-acetyl group
PCP	phenol-chloroform-petroleum (extraction)
ZIC^®^HILIC	zwitterionic-hydrophilic interaction liquid chromatography

## References

[1] Kunin C.M., Beard M.V., Halmagyi N.E. (1962). Evidence for a common hapten associated with endotoxin fractions of *E. coli* and other *Enterobacteriaceae*. Exp. Biol. Med..

[2] Bottger E.C., Jurs M., Barrett T., Wachsmuth K., Metzger S., Bitter-Suermann D. (1987). Qualitative and quantitative determination of enterobacterial common antigen (ECA) with monoclonal antibodies: Expression of ECA by two *Actinobacillus* species. J. Clin. Microbiol..

[3] Rai A.K., Mitchell A.M. (2020). Enterobacterial common antigen: Aynthesis and function of an enigmatic molecule. mBio.

[4] Lugowski C., Romanowska E., Kenne L., Lindberg B. (1983). Identification of a trisaccharide repeating-unit in the enterobacterial common-antigen. Carbohydr. Res..

[5] Vinogradov E.V., Knirel Y.A., Thomas-Oates J.E., Shashkov A.S., L’Vov V.L. (1994). The structure of the cyclic enterobacterial common antigen (ECA) from Yersinia pestis. Carbohydr. Res..

[6] Kajimura J., Rahman A., Hsu J., Evans M.R., Gardner K.H., Rick P.D. (2006). O acetylation of the enterobacterial common antigen polysaccharide is catalyzed by the product of the yiaH gene of *Escherichia coli* K-12. J. Bacteriol..

[7] Kiss P., Rinno J., Schmidt G., Mayer H. (1978). Structural studies on the immunogenic form of the enterobacterial common antigen. Eur. J. Biochem..

[8] Dell A., Oates J., Lugowski C., Romanowska E., Kenne L., Lindberg B. (1984). The enterobacterial common-antigen, a cyclic polysaccharide. Carbohydr. Res..

[9] Rick P.D., Hubbard G.L., Kitaoka M., Nagaki H., Kinoshita T., Dowd S., Simplaceanu V., Ho C. (1998). Characterization of the lipid-carrier involved in the synthesis of enterobacterial common antigen (ECA) and identification of a novel phosphoglyceride in a mutant of *Salmonella typhimurium* defective in ECA synthesis. Glycobiology.

[10] De Vlugt J.E., Xiao P., Munro R., Charchoglyan A., Brewer D., Al-Abdul-Wahid M.S., Brown L.S., Ladizhansky V. (2020). Identifying lipids tightly bound to an integral membrane protein. Biochim. Biophys. Acta Biomembr..

[11] Gozdziewicz T.K., Lugowski C., Lukasiewicz J. (2014). First evidence for a covalent linkage between enterobacterial common antigen and lipopolysaccharide in *Shigella sonnei* phase II ECALPS. J. Biol. Chem..

[12] Maciejewska A., Kaszowska M., Jachymek W., Lugowski C., Lukasiewicz J. (2020). Lipopolysaccharide-linked enterobacterial common antigen (ECA(LPS)) occurs in rough strains of *Escherichia coli* R1, R2, and R4. Int. J. Mol. Sci..

[13] Kunin C.M. (1963). Separation, characterization, and biological significance of a common antigen in *Enterobacteriaceae*. J. Exp. Med..

[14] Kunin C.M., Beard M.V. (1963). Serological studies of O antigens of *Escherichia coli* by means of the hemagglutination test. J. Bacteriol..

[15] Mitchell A.M., Srikumar T., Silhavy T.J. (2018). Cyclic enterobacterial common antigen maintains the outer membrane permeability barrier of *Escherichia coli* in a manner controlled by YhdP. mBio.

[16] Muszynski A., Rabsztyn K., Knapska K., Duda K.A., Duda-Grychtol K., Kasperkiewicz K., Radziejewska-Lebrecht J., Holst O., Skurnik M. (2013). Enterobacterial common antigen and O-specific polysaccharide coexist in the lipopolysaccharide of *Yersinia enterocolitica* serotype O:3. Microbiology.

[17] Paunova-Krasteva T.S., Pavlova V.A., De Castro C., Ivanova R.M., Molinaro A., Nikolova E.B., Stoitsova S.R. (2014). Cyclic enterobacterial common antigens from *Escherichia coli* O157 as microbe-associated molecular patterns. Can. J. Microbiol..

[18] Liu L., Zha J., DiGiandomenico A., McAllister D., Stover C.K., Wang Q., Boons G.J. (2015). Synthetic enterobacterial common antigen (ECA) for the development of a universal immunotherapy for drug-resistant *Enterobacteriaceae*. Angew. Chem. Int. Ed. Engl..

[19] Mannel D., Mayer H. (1978). Isolation and chemical characterization of the enterobacterial common antigen. Eur. J. Biochem..

[20] Lugowski C., Romanowska E. (1978). Enterobacterial common antigen: Isolation from *Shigella sonnei*, purification and immunochemical characterization. Eur. J. Biochem..

[21] Peters H., Jurs M., Jann B., Jann K., Timmis K.N., Bitter-Suermann D. (1985). Monoclonal antibodies to enterobacterial common antigen and to *Escherichia coli* lipopolysaccharide outer core: Demonstration of an antigenic determinant shared by enterobacterial common antigen and E. coli K5 capsular polysaccharide. Infect. Immun..

[22] Kuhn H.M., Basu S., Mayer H. (1987). Comparison of enterobacterial common antigen from different species by serological techniques. Eur. J. Biochem..

[23] Kuhn H.M., Neter E., Mayer H. (1983). Modification of the lipid moiety of the enterobacterial common antigen by the “*Pseudomonas* factor”. Infect. Immun..

[24] Farnback M., Eriksson L., Senchenkova S., Zych K., Knirel Y.A., Sidorczyk Z., Widmalm G. (2003). Crystal structure of a cyclic enterobacterial common antigen. Angew. Chem. Int. Ed. Engl..

[25] Fregolino E., Ivanova R., Lanzetta R., Molinaro A., Parrilli M., Paunova-Krasteva T., Stoitsova S.R., De Castro C. (2012). Occurrence and structure of cyclic enterobacterial common antigen in *Escherichia coli* O157:H(-). Carbohydr. Res..

[26] Westphal O. (1965). Bacterial lipopolysaccharides extraction with phenol-water and further applications of the procedure. Met. Carbohydr. Chem..

[27] Duda K.A., Duda K.T., Beczala A., Kasperkiewicz K., Radziejewska-Lebrecht J., Skurnik M. (2009). ECA-immunogenicity of *Proteus mirabilis* strains. Arch. Immunol. Ther. Exp..

[28] Weber G., von Wirén N., Hayen H. (2008). Hydrophilic interaction chromatography of small metal species in plants using sulfobetaine- and phosphorylcholine-type zwitterionic stationary phases. J. Sep. Sci..

[29] Man-Kupisinska A., Bobko E., Gozdziewicz T.K., Maciejewska A., Jachymek W., Lugowski C., Lukasiewicz J. (2016). Fractionation and analysis of lipopolysaccharide-derived oligosaccharides by zwitterionic-type hydrophilic interaction liquid chromatography coupled with electrospray ionisation mass spectrometry. Carbohydr. Res..

[30] Ceroni A., Maass K., Geyer H., Geyer R., Dell A., Haslam S.M. (2008). GlycoWorkbench: A tool for the computer-assisted annotation of mass spectra of glycans. J. Proteome Res..

